# Neuron-derived CCL2 contributes to microglia activation and neurological decline in hepatic encephalopathy

**DOI:** 10.1186/s40659-017-0130-y

**Published:** 2017-09-04

**Authors:** Li Zhang, Jinyun Tan, Xiaoping Jiang, Weiwei Qian, Ting Yang, Xijun Sun, Zhaohui Chen, Qiwen Zhu

**Affiliations:** Department of Radiology, The Second People’s Hospital of Lanzhou, No. 388 Jingyuan Road, Chengguan District, Lanzhou, 730046 China

**Keywords:** Hepatic encephalopathy, Neuron, Microglia, Chemokine CC motif ligand 2

## Abstract

**Background:**

CCL2 was up-regulated in neurons and involved in microglia activation and neurological decline in mice suffering from hepatic encephalopathy (HE). However, no data exist concerning the effect of neuron-derived CCL2 on microglia activation in vitro.

**Methods:**

The rats were pretreated with CCL2 receptor inhibitors (INCB or C021, 1 mg/kg/day *i.p.*) for 3 days prior to thioacetamide (TAA) administration (300 mg/kg/day *i.p.*) for inducing HE model. At 8 h following the last injection (and every 4 h after), the grade of encephalopathy was assessed. Blood and whole brains were collected at coma for measuring CCL2 and Iba1 expression. In vitro, primary neurons were stimulated with TNF-α, and then the medium were collected for addition to microglia cultures with or without INCB or C021 pretreatment. The effect of the medium on microglia proliferation and activation was evaluated after 24 h.

**Results:**

CCL2 expression and microglia activation were elevated in the cerebral cortex of rats received TAA alone. CCL2 receptors inhibition improved neurological score and reduced cortical microglia activation. In vitro, TNF-α treatment induced CCL2 release by neurons. Medium from TNF-α stimulated neurons caused microglia proliferation and M1 markers expression, including iNOS, COX2, IL-6 and IL-1β, which could be suppressed by INCB or C021 pretreatment. The medium could also facilitate p65 nuclear translocation and IκBα phosphorylation, and NF-κB inhibition reduced the increased IL-6 and IL-1β expression induced by the medium.

**Conclusion:**

Neuron-derived CCL2 contributed to microglia activation and neurological decline in HE. Blocking CCL2 or inhibiting microglia excessive activation may be potential strategies for HE.

## Background

Hepatic encephalopathy (HE) is a dangerous neuropsychiatric complication of both acute and chronic liver failure, and is the most common cause of death in patients with end-stage liver disease. The clinical symptom of HE included disturbance of consciousness, abnormal behavior, and coma. At present, the pathogenesis of HE has not been fully clarified, and there is no efficient approaches for controlling HE, so it has long been a hot topic area worldwide.

The main neuropathological features of HE were morphological and functional changes of glial cells [1]. Microglia is the important immune cells in the central nervous system (CNS), and distributed in the whole brain and retina. About 12% of adult brain cells are microglia, which plays an important role in innate immune or inflammatory responses. Microglia activation has been repeatedly reported in numerous rodent models of HE, and in patients suffering from HE [2–4]. Excessive activated microglia release a large number of inflammatory cytokines such as IL-6, NO, IL-1β, TNF-α, and the accumulation of these inflammatory factors leads to the neurotoxicity.

Chemokine CC motif ligand 2 (CCL2) also known as monocyte chemoattractant protein-1 (MCP-1), is produced by various cell types in the brain, such as neurons, astrocyte and microglia [5, 6]. A substantial body of evidence exists suggesting CCL2 is involved in many neuroinflammation and neurodegenerative diseases. Recently, it was demonstrated expression of CCL2 in neurons were appreciably raised in mice with HE, which resulted in microglia activation and neurological dysfunction [7]. However, a previous study by Ara E Hinojosa et al. showed CCL2 was not able to induce microglial activation, either by itself or in combination with LPS, and could not induce cell death of neurons co-cultured with microglia [8], suggesting other factors may be necessary to cause the changes that result in the neuronal damage commonly observed in situations where CCL2 levels are elevated.

In this work we found condition medium of neurons stimulated with TNF-α, with high level of CCL2, could promote microglia activation, which could be suppressed by the blockage of CCL2 receptors. Combined with some published results [8], the present study indicated that some other factors derived from neurons may cooperate with CCL2 to induce microglia activation during some pathological conditions, including HE. However, which factors are involved in this process needs further investigation.

## Methods

### Rat model of hepatic encephalopathy

All the animal procedures were approved by the Ethics Committee of the Second People’s Hospital of Lanzhou. Forty male SD rats (Shanghai SLAC laboratory animal Co., Ltd., Shanghai, China), weighting 180 ~ 220 g, were randomly divided into 4 groups: vehicle, TAA, TAA + INCB and TAA + C021 group. The rats in TAA + INCB and TAA + C021 groups were pretreated with INCB (1 mg/kg/day *i.p.*) and C021 (1 mg/kg/day *i.p.*) for 3 days prior to TAA administration [7]. Then, the three groups (except vehicle group) were given intraperitoneal injection of 300 mg/kg/day thioacetamide (TAA) for three days to establish hepatic encephalopathy (HE) model. Following injection, rats were placed on heating pads adjusted to 37 °C to control temperature. To avoid hypoglycemia and dehydration, after 12 h and every subsequent 4 h, the rats were subcutaneously administered with 0.5 ml/kg of fluid therapy (5% dextrose and 0.45% saline containing 20 mEq/l of potassiumchloride). Rats in vehicle group were treated with saline of the same volume. At 8 h following the last injection (and every 4 h after), encephalopathy grade of rats was assessed according to the following grade standards for brain function: 0, normal; 1, drowsiness, slow response, decreased autonomic activity, normal reflex; 2, ataxia, basically normal reflex; 3, abnormal reflex; 4, loss of corneal reflex, coma. Blood, liver and whole brains were collected at coma and frozen for further analysis.

### Liver histology and biochemistry

Liver tissue specimens procured from the left lateral hepatic lobe were fixed with 10% neutral formaldehyde solution and embedded with paraffin. Then, the tissue specimens were sectioned into 3 μm sections and stained with hematoxylin and eosin (HE) using standard procedures. The slides were viewed and imaged using an Olympus BX40 microscope with Olympus DP25 imaging system (Olympus, USA). Serum alanine aminotransferase (ALT) and bilirubin were determined using commercially available kits (Nanjing Jiancheng, China) according to manufacturers’ instructions.

### Immuno-histochemical analysis

The cerebral cortex was fixed in 10% formalin, embedded in paraffin, and cut into 3 μm sections. After dewaxing, dehydration and antigen retrieval, the sections were incubated with 3% hydrogen peroxide solution at room temperature for 25 min to quench endogenous peroxidase. After blocked by 3% BSA, the sections were incubated with anti-Iba1 antibody (1:8000, ab178847, Abcam) at 4 °C for 24 h. Then, the sections were incubated at room temperature for 45 min with secondary antibodies labeled with horseradish peroxidase (HRP). Afterwards, the slides were incubated in the chromogen 3,3′ -diaminobenzidine (DAB; Vector) at room temperature and coloration time was controlled through a microscope. Sections were then washed in running tap water, counterstained with hematoxylin, and mounted. Images were taken using an inverted microscope (Nikon, Japan).

### Neurons cultures and TNF-α stimulation

Neonatal SD rats, 1 day-old, were decapitated and the brain tissue was removed under sterile condition. The cerebral cortex was immediately dissected and placed in the precooling D-hanks buffered saline. After removal of meninges and blood vessels, the cerebral cortex was shredded and digested with 0.1% trypsin for 20 min at 37 °C. After terminating the digestion with a few drops of FBS, neurons were mechanically dissociated in DMEM/F12 supplemented with 5% FBS, seeded at 1 × 10^5^ cells/ml in poly-l-lysine-precoated 24 holes plate and cultured at 37 °C, 5% CO_2_ conditions in the incubator. After 12 h, the medium was replaced with serum-free neuron basal/B27 medium supplemented with 0.5 mmol/l glutamine. After 3 days 50% of the medium was replaced with fresh Neuron basal medium. The purity of the neuron was >92% as determined by NeuN antibody.

The cultured neurons were treated with 0.1, 1 or 10 ng/ml TNF-α for 15 min or 10 ng/ml TNF-α for 5, 10 and 20 min. Then the cells were washed with PBS and cultured in fresh medium. After 3 h, CCL2 mRNA expression and concentration in culture medium were determined with qRT-PCR and ELISA assays. The culture medium and cell lysates were collected for subsequent addition to microglia cultures.

### Microglia cultures and treatments

The primary cortical microglial cells were prepared from 8 neonatal SD rats (1–2 days old). Briefly, the rats were sacrificed under anaesthesia and the brains were removed. After removal of meninges and other non-cortical tissues with dissecting forceps, the remaining cortexes were moved rapidly into the precooling HBSS. After removal of midbrain and hippocampus with scalpel, the separated cortexes were digested with 2 ml 0.25% trypsin for 12 min at 37 °C. After digestion, cells were cultured in T-75-cm^2^ flasks in MEM supplemented with 10% FBS and antibiotics for 10 days. When the cells reached about 98% confluence, the microglial cells were mechanically separated from the mixed cells by mild trypsinization and brief shaking. The culture medium was centrifuged by 2800 r/min for 5 min. Then the isolated microglial cells were resuspended in DMEM supplemented with 0.1% FBS, inoculated in 24 pore culture plate and cultured at 37 °C. The primary microglial cells were identified with immunofluorescence analysis using an anti-CD11b/c antibody, and only the microglial cells of at least 90% purity were adopted in this work.

The cultured microglia were divided into 4 groups: Ctrl, receiving culture medium (CM) from neurons; CM, receiving CM from neurons stimulated with TNF-α; CM + INCB, the cells were pretreated with 5 μM INCB (CCR2 inhibitor) for 1 h before administered with CM from neurons stimulated with TNF-α; CM + C021, the cells were pretreated with 5 μM C021 (CCR4 inhibitor) for 1 h before administered with CM from neurons stimulated with TNF-α. After cultured for 24 h, the cell proliferation was evaluated with the Cell Counting Kit-8 (Dojindo Laboratories, Japan) [9], and the expression of Iba1, iNOS, COX2, Arg-1 and YM-1, and secretion of IL-6, IL-1β, IL-4 and IL-10 by microglia were determined by qRT-PCR, western blotting and ELISA assays.

### RNA isolation and qRT-PCR

RNA was prepared from flash frozen tissue and cells using TRIZOL^©^ reagent (invitrogen) in accordance with manufacturer’s instructions, and then reverse-transcribed to cDNA using random hexamer primers. The qRT-PCR was carried out using SYBR^®^ Green Real time PCR Master Mix (Toyobo Co. Ltd., Osaka, Japan) using the following conditions: 94 °C for 5 min, and followed by 40 cycles of 94 °C for 30 s, 58–61 °C for 30 s depending on the primers, and 72 °C for 2 min. The mRNA level of β-actin was used as an internal control, and the relative gene expression levels were calculated using the 2^−ΔΔCt^ method. Each gene was analyzed in triplicate. The primer sequences were listed as follows: CCL2, F 5′-GCTCATAGCAGCCACC TCATTC-3′ and R 5′-CCGCCAAAATAACCGATGTGATAC-3′; Iba1, F 5′-CTCCTCCAAGGC CCAAACTA-3′and R 5′-AAAC CAAGGATGCGGTAGACA-3′; iNOS, F 5′-GGGAGCCAGA GCAGTACAAG-3′ and R 5′-TGCAGATTCTGGAGGGATTT-3′; COX2, F 5′-GAAGTCTTTGG TCTG GTGCCT-3′ and R 5′-GCAATGCGGTTCTGATACTGG-3′; Arg-1, F 5′-TTAGGCCAAG GTGCTTGCTGCC-3′and R 5′-TACCATGGCCCTGAGGAGGTTC-3′; YM-1, F 5′-GGGCATA CCTTTATCCTGAG-3′ and R 5′-TGAAGTCATCCATGTC-3′; IL-6, F 5′-AACTCCATCTGCCC TTCAGGAACA-3′and R 5′-AAGGCAGTGGCT GTCA ACAACATC-3′; IL-1β, F 5′-ACCTGC TAGT GTGTGATGTTCCCA-3′ and R 5′-AG GTGGAGAGCTTTCAGCTCACAT-3′; β-actin, F 5′-AGATCCTGACCGAGCGTGGC-3′ and R 5′-CCAGGGAGGAAGAGGATGCG-3′.

### Western blotting analysis

The tissue samples were ground under liquid nitrogen and the cell samples were washed with Hank’s balanced salt. Then, the samples were lysed in ice-cold RIPA lysis buffer (Beyotime Inc., Nanjing, China) in the presence of protease inhibitors (Sigma, P8349) and incubated on ice for 40 min. For determination of NF-κB signal pathway, the cytoplasmic and nuclear proteins were extracted from the primary cortical microglial cells using the Nuclear and Cytoplasmic Protein Extraction Kit (Beyotime Biotechnology, China). Total protein content was measured using a BCA protein assay kit (Applygen, China). Then the proteins were separated by SDS-PAGE using 10% gels and transferred onto PVDF membranes (Thermo Fisher Scientific, USA). After blocking for 30 min at room temp in blocking solution containing 5% non-fat milk, the membranes were incubated overnight at 4 °C with primary antibody: anti-Iba1 antibody (1:1000, ab178847, Abcam); NF-κB p65 (1:50,000, ab32536, Abcam), IκBα (1:1000, ab109300, Abcam) and pIκBα (1:1000, ab12135, Abcam). After several washes, the membranes were incubated with an appropriate HRP-conjugated secondary antibody for 1 h at room temperature. The proteins bands were visualized using ECL kits (Amersham) and the optical density of the protein bands was quantified using the ImageJ software. Beta-actin was used as an internal control.

### ELISA assays

CCL2 concentration in cerebral cortex and serum, and the secretion of CCL2, IL-6, IL-1β, IL-4 and IL-10 by the primary cortical microglial cells were measured using ELISA kits (R&D Systems, USA) according to the manufacturer’s instructions.

### Statistical analysis

All data were presented as mean ± standard deviation (SD). Multiple comparisons were performed using one way ANOVA followed by Duncan’s post hoc test, and *P* < 0.05 or *P* < 0.01 was considered as statistically significant. All assays were performed in triplicate.

## Results

### CCL2 blockage alleviated TAA-induced neurological decline and liver damage

**Fig. 1:**
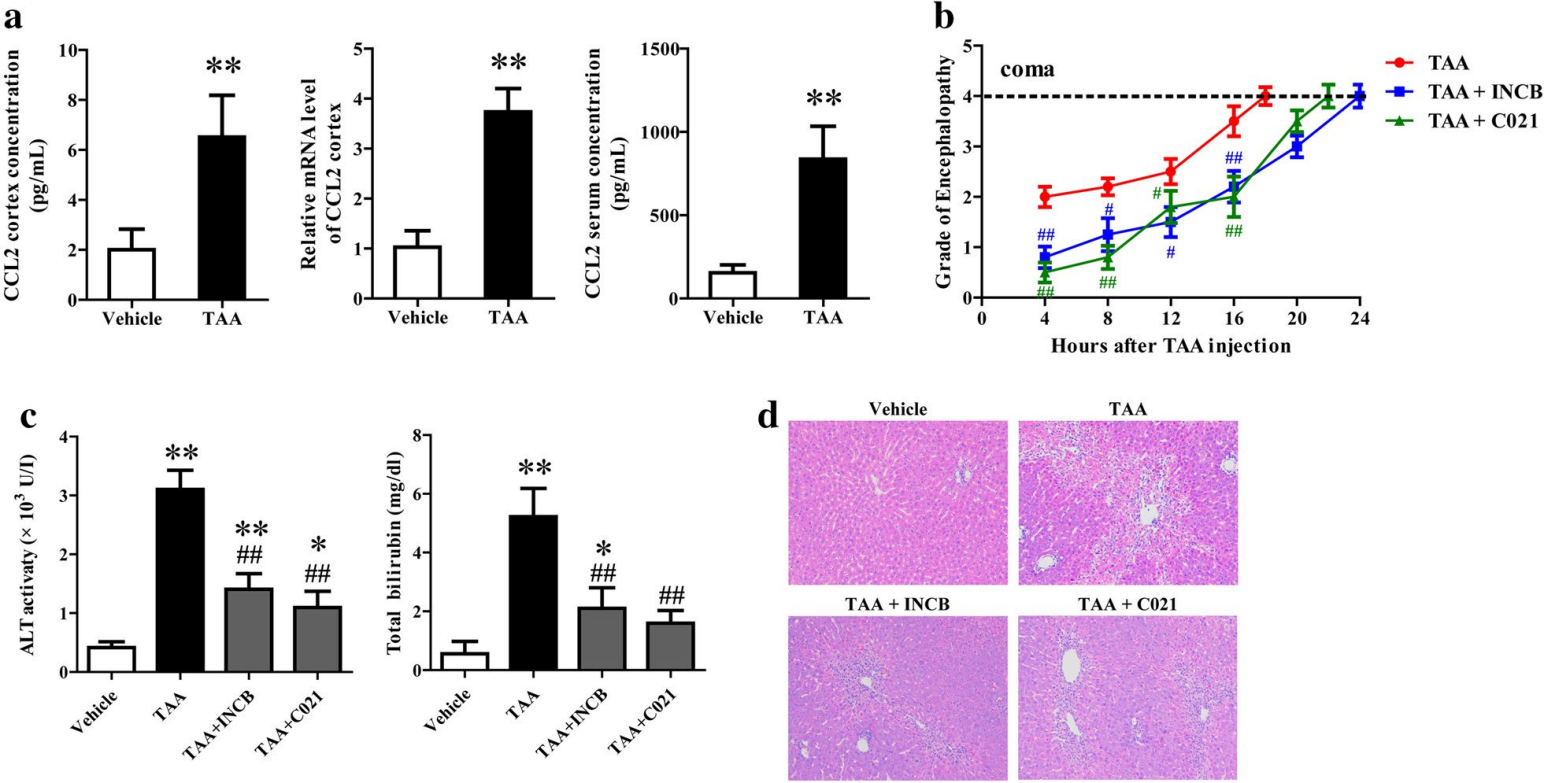
CCL2 blockage alleviated TAA-induced neurological decline and liver damage. At 8 h following the last injection of thioacetamide (TAA), and every 4 h after, the encephalopathy grade of rats from different group was assessed (**b**). Blood, whole brains and liver tissue were collected at coma and CCL2 expression and concentration in cerebral cortex and serum were determined using qRT-PCR and Elisa (**a**). Liver function was determined through measuring the serum levels of ALT and total bilirubin using commercially available kits (**c**). Liver damage was evaluated using HE staining and the original magnification was ×100 (**d**). **P < 0.01, *P < 0.05, vs vehicle; ^##^ *P* < 0.01, ^#^ *P* < 0.05, vs TAA

CCL2 plays an important role in mediating inflammatory response in various neurodegenerative diseases. The expression and concentration of CCL2 in cerebral cortex and serum from rats treated with TAA were significantly higher than that from control rats (Fig. 1a), which was in accord with a previous study [7]. After confirmation of CCL2 upregulation in HE rat model, we intended to explore the contribution of CCL2 in TAA induced liver damage and neurological decline through injection of CCL2 receptors (CCR2 and CCR4) inhibitors (INCB or C021) into rats. CCL2 blockage considerably reduced the grade of encephalopathy and extended the time to coma, indicating the elevated CCL2 after TAA treatment contributes to the occurrence and development of neurological decline (Fig. 1b). Analyses of circulating levels of ALT and total bilirubin, indicative of liver function, demonstrated pretreatment with either CCL2 receptor antagonists significantly reduced TAA induced elevation of ALT and bilirubin, though levels were still higher than controls (Fig. 1c). Liver histological examination showed although TAA induced liver damage was obviously ameliorated by either INCB or C021 pretreatment, significant necrosis and steatosis were still present in the liver tissues (Fig. 1d).

### CCL2 blockage alleviated TAA-induced microglia activation

**Fig. 2:**
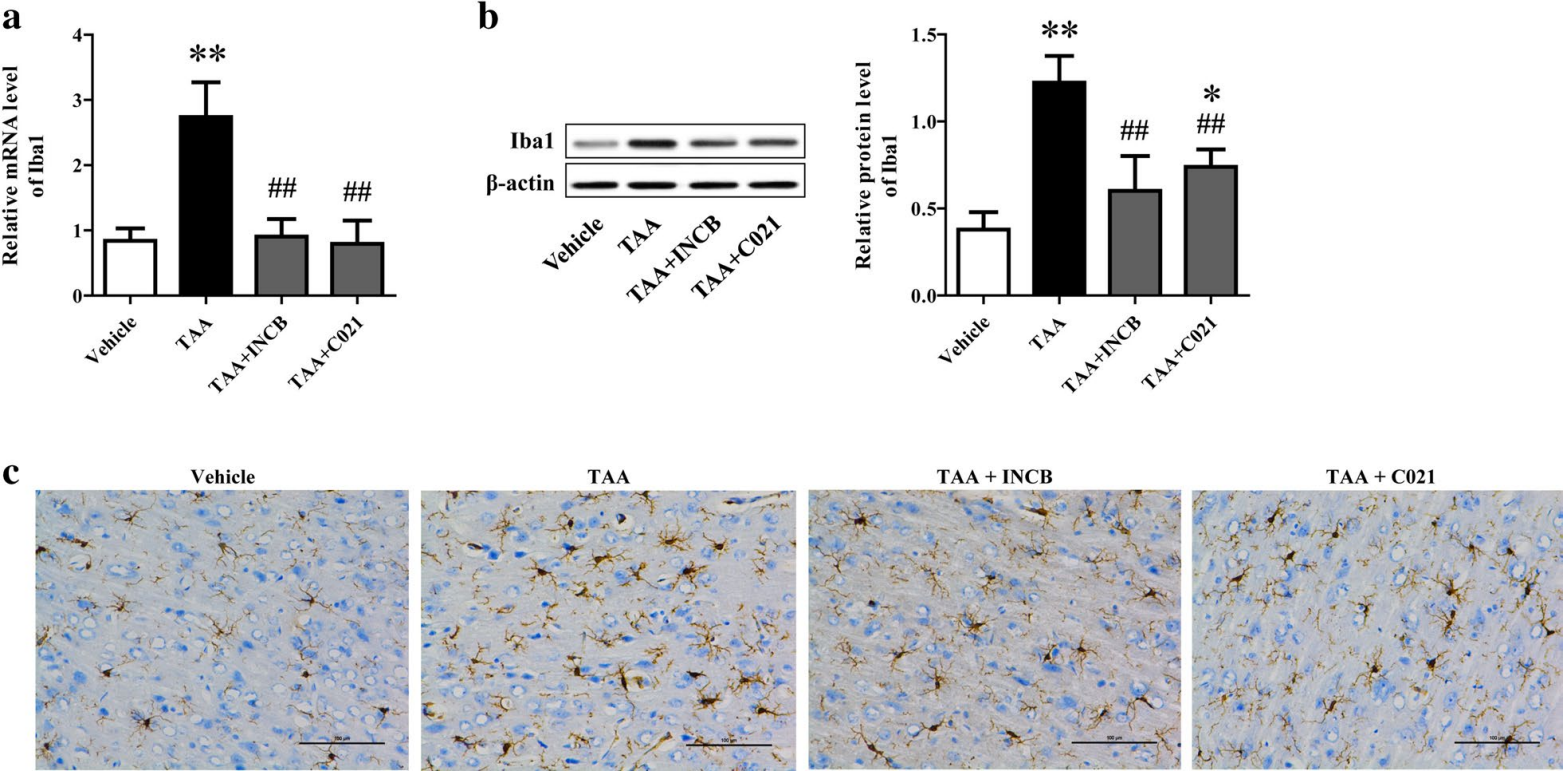
CCL2 blockage alleviated TAA-induced microglia activation. Microglia activation in cerebral cortex collected at coma were evaluated through determination of Iba1 expression, a marker of microglia activation, using qRT-PCR (**a**), western blotting (**b**) and immunohistochemistry assays (**c**). The original magnification for immunohistochemical picture was ×200. **P < 0.01, *P < 0.05, vs vehicle; ^##^ *P* < 0.01, ^#^ *P* < 0.05, vs TAA

Numerous studies have demonstrated that increased microglia activation and subsequent neuroinflammation contributed to the liver damage induced neurological decline and coma in HE [2, 10]. In addition, CCL2 could induce the activation of microglia in various neuropathies and lead to a proinflammatory response. Thus, microglia activation was assessed in the brains through determination of Iba1 expression. The results of qRT-PCR and western blot analysis showed Iba1 expression in the cerebral cortex was markedly elevated by TAA treatment, which was remarkably reduced through inhibition of CCR2 or CCR4 (Fig. 2a, b), indicating elevated CCL2 level after TAA treatment resulted in increased microglia activation. To address whether the increase in Iba1 is due to more microglia recruitment, or a more activated phenotype of existing microglia, or both, immunohistochemistry for Iba1 was performed. The results identified that compared to vehicle-treated mice, TAA-treated mice showed a significantly increased microglia aggregation, and the individual microglia had a more amoeboid appearance (Fig. 2c). Pretreatment of mice with the antagonists against CCR2 and CCR4 significantly prevented microglia recruitment and the full activation of microglia, though partially activated and quiescent microglia were presented in these rats (Fig. 2c).

### TNF-α induced CCL2 expression and release in cultured neurons

**Fig. 3:**
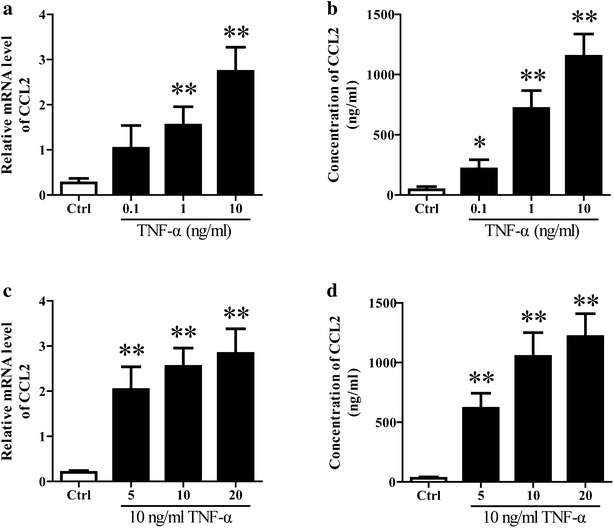
TNF-α stimulation induced CCL2 expression and release in cultured neurons. The primary neurons isolated from neonatal rats were stimulated with different concentrations of TNF-α (0.1, 1 or 10 ng/ml) for 15 min or 10 ng/ml of TNF-α for 5, 10 or 20 min. Then the cells were washed with PBS and cultured in fresh medium. After 3 h, the CCL2 mRNA expression (**a**, **c**) and content in medium (**b**, **d**) were determined using qRT-PCR and ELISA respectively. **P < 0.01, *P < 0.05, vs Ctrl

The results of in vivo experiments suggested elevated CCL2 contributed to TAA induced microglia activation and subsequent neurological decline in rats suffering from HE. However, the source of CCL2 was still unknown. Past findings have suggested that CCL2 is expressed by multiple cell types in the CNS including neurons, microglia, endothelial cells, and astrocytes [11–13]. In particular, neurons are the primary source of CCL2 during HE in mice [7]. Thus, we speculated neurons might also be the main source of CCL2 in rats with HE, at least in the brain tissue. To test the potential of neurons being the source of CCL2 during HE, we stimulated neurons with TNF-α to mimic the HE condition [14], and CCL2 mRNA expression and release was measured. The cultured neurons were stimulated with different concentrations of TNF-α (0.1, 1 or 10 ng/ml) for 15 min or 10 ng/ml of TNF-α for 5, 10 or 20 min. Then the cells were washed with PBS and cultured in fresh medium. After 3 h, CCL2 expression and content in medium were determined using qRT-PCR and ELISA respectively. The results showed TNF-α stimulation induced CCL2 expression and secretion in neurons in a time- and dose-dependent (Fig. 3).

### Neuron-derived CCL2 induced the proliferation and classical activation of microglia

**Fig. 4:**
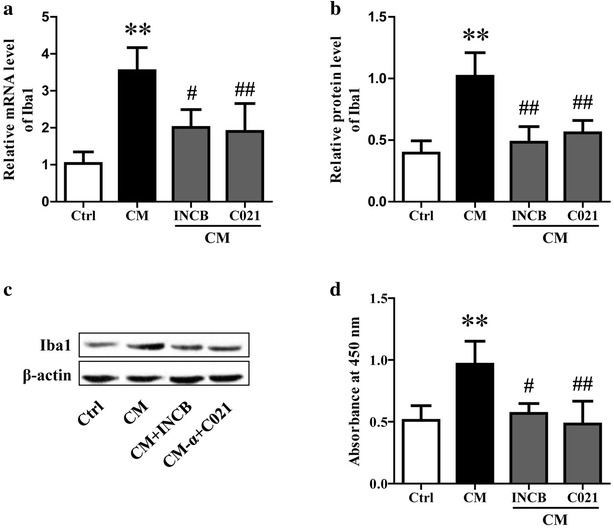
Neuron-derived CCL2 induced the proliferation and activation of microglia. After stimulation with 10 ng/ml TNF-α for 10 min and washed with PBS, neurons were cultured in fresh medium for 3 h. Then the medium and lysates (*CM*) were collected and added into microglia cultures. To block CCL2, the microglia was pretreated with 5 μM CCL2 receptor inhibitor (INCB or C021) for 1 h before the addition of CM. After incubation for 24 h, the expression of Iba1 was determined using qRT-PCR (**a**) and western blotting (**b**, **c**). The proliferation of microglia was measured using CCK8 assay (**d**). **P < 0.01, *P < 0.05, vs Ctrl; ^##^ *P* < 0.01, ^#^ *P* < 0.05, vs CM

Based on the obtained experimental results, we speculated that CCL2 derived from neurons led to the activation of microglia during HE. However, an existing study showed CCL2 alone was not able to induce microglial activation and cell death of neurons co-cultured with microglia [8], suggesting some other factors may be necessary to cause the changes that result in the neuronal damage commonly observed in situations where CCL2 levels are elevated. To test this hypothesis, we added the condition medium of neurons stimulated with TNF-α into microglia cultures. After incubation for 24 h, the proliferation and activation of microglia were assayed using CCK8, qRT-PCR and western blotting methods. To block CCL2, the microglia were pretreated with 5 μM CCL2 receptor inhibitor (INCB or C021) for 1 h before the addition of collected medium and lysates. The results showed medium from neurons stimulated with TNF-α significantly improved the mRNA and protein expression of Iba1 in microglia, which could be observably restrained by INCB or C021 pretreatment (Fig. 4a–c). The results indicated neuron-derived CCL2 plays a critical role in microglia activation. In addition, microglia proliferation was also promoted by medium from TNF-α stimulated neurons (Fig. 4d).

**Fig. 5:**
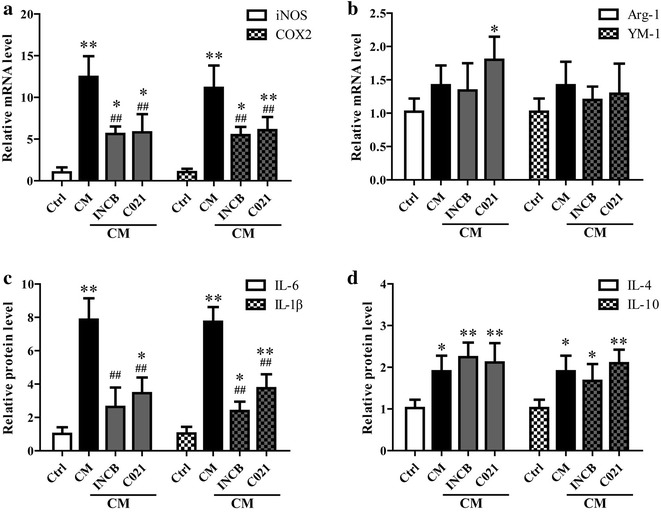
Neuron-derived CCL2 induced the expression of M1 markers in microglia. As mentioned above, the medium and lysates (*CM*) of neurons stimulated with TNF-α were collected and added into microglia cultures. The microglia was pretreated with 5 μM CCL2 receptor inhibitor (INCB or C021) for 1 h before the addition of CM to block CCL2. After incubation for 24 h, the mRNA expression of iNOS, COX2, Arg-1 and YM-1 in microglia was determined using qRT-PCR (**a**, **b**), and the release of IL-6, IL-1, IL-1β, IL-4 and IL-10 were determined using Elisa assay (**c**, **d**). **P < 0.01, *P < 0.05, vs Ctrl; ^##^ *P* < 0.01, ^#^ *P* < 0.05, vs CM

The activated microglia is usually divided into two types, “classically activated” M1 and “alternatively activated” M2 microglia. M2 microglia is involved in tissue repair and neurons regeneration. However, M1 microglia is usually with cytotoxic properties. The fact that neuron-derived CCL2 induced microglia activation prompted us to determine the expression of M1 and M2 markers in microglia. The results showed mRNA levels of iNOS and COX2, and the release of IL-6 and IL-1β were significantly increased in microglia stimulated with medium from neurons treated with TNF-α, which could be suppressed by INCB or C021 pretreatment (Fig. 5a, c). Conversely, no significant changes were observed in the mRNA expression of Arg-1 and YM-1 (Fig. 5b). Although the release of IL-4 and IL-10 was increased after CM treatment, CCL2 blockage showed little influence on the release of both IL-4 and IL-10 (Fig. 5d), suggesting the increased production of IL-4 and IL-10 was not due to CCL2 stimulation. These results suggested neuron-derived CCL2 induces microglia classical activation (M1).

### Neuron-derived CCL2 stimulated the activation of NF-κB in microglia

**Fig. 6:**
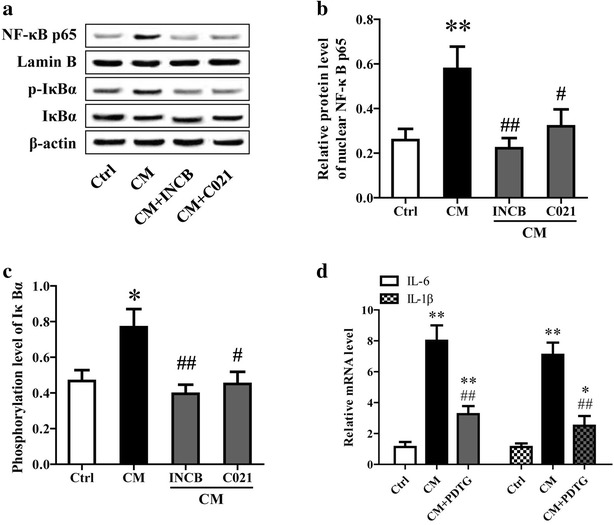
Neuron-derived CCL2 stimulated the activation of NF-κB in microglia. As previously described, the medium and lysates (*CM*) of neurons stimulated with TNF-α were collected and added into microglia cultures. The microglia was pretreated with 5 μM CCL2 receptor inhibitor (INCB or C021) for 1 h before the addition of CM to block CCL2. After incubation for 24 h, NF-κB activation was evaluated through determination of IκBα phosphorylation and NF-κB p65 nuclear translocation using western blotting (**a**–**c**). **d** The cultured microglia was pretreated with 10 μM PDTG (NF-κB inhibitor) for 1 h prior to administration of CM. After cultured for 24 h, the mRNA expression of IL-6 and IL-1β was measured using qRT-PCR. **P < 0.01, *P < 0.05, vs Ctrl; ^##^ *P* < 0.01, ^#^ *P* < 0.05, vs CM

NF-κB is a classical nuclear transcription factor related to inflammation, which plays an important role in the occurrence and progression of inflammatory response. To investigate the effect of neuron-derived CCL2 on NF-κB activation in microglia, the cytoplasmic and nuclear proteins were prepared from the cultured microglial cells. The protein level of nuclear NF-κB p65 and phosphorylation level of IκBα in cytoplasm were determined using western blotting analysis. The results showed medium from neurons stimulated with TNF-α could significantly facilitate NF-κB subunit p65 nuclear translocation and IκBα phosphorylation, and INCB or C021 pretreatment could remarkably restrain this promoting effect (Fig. 6a–c). Thus, we hypothesized that neuron-derived CCL2 could promote the expression of iNOS, COX-2, IL-6 and IL-1β through facilitating NF-κB activation.

To test this, the cultured microglia was pretreated with 10 μM PDTG (NF-κB inhibitor) for 1 h prior to administration of medium from neurons. After cultured for 24 h, the mRNA expression of IL-6 and IL-1β in microglia was measured using qRT-PCR. The results showed pretreatment with PDTG significantly attenuated the increased expression of IL-6 and IL-1β induced by medium from neurons treated with TNF-L (Fig. 6d). Together, these data indicated that neuron-derived CCL2 contributes microglia activation and release of inflammatory factors through NF-κB pathway.

## Discussion

In the present study we confirmed that CCL2 expression and microglia activation in cerebral cortex were significantly increased in the rat model of HE, and inhibition of CCL2 receptors (CCR2 or CCR4) could notably reduce the microglia activation and neurological decline in rats with HE. In addition, we demonstrated neuron-derived CCL2 contributed to microglia proliferation, classical activation (M1) and release of inflammatory factor. The underlying mechanism was related to the activation of NF-κB signal pathway.

HE is a complication of severe liver failure, and its pathogenesis is not completely clear. But, it was recommended proinflammatory cytokines released by activated microglia were involved [4]. Under normal conditions, activated microglia play a neuroprotective role through release of neurotrophic factors, phagocytosis of injured nerve cells and inducing tissue repair [15]. However, excessively activated microglia produce a large number of inflammatory cytokines such as IL-6, NO, IL-1β, TNF-α, which are toxic for nerve cells [15, 16]. Microglia activation and concomitantly increased release of proinflammatory cytokines have been repeatedly observed in various animal models of HE induced by surgery or hepatotoxic agents [4, 7, 10, 17, 18]. In addition, microglia activation was also upregulated in patients with HE, including cirrhotic patients, alcoholic patients and viral hepatitis patients [7]. Moreover, the prevention of microglia activation or inflammatory response using minocycline, hypothermia, N-acetylcysteine, etanercept, ibuprofen and indomethacin could significantly attenuate neurological function decline and brain edema in HE [19, 20]. In this study, increased activation of microglia was observed in rats with HE, and inhibition of microglia activation significantly reduced the grade of encephalopathy, which were consistent with previous studies, indicating microglia activation plays an important role in the occurrence and development of HE.

Multiple signaling mechanisms of microglia activation during HE have been proposed. In addition to toxic metabolites generated by the failing liver such as ammonia, lactate, glutamate, manganese, and neurosteroids, blood–brain cytokine transfer and receptor-mediated cytokine signal transduction may also play a role in the microglia activation during HE [21]. It was found that peripheral TNF-α could indirectly cause microglia activation and subsequent recruitment of monocytes into the brain and in situ production of TNF-α through stimulating the activation of cerebrovascular endothelial cells in an animal model of biliary cirrhosis [17]. In line with this, it has been showed treatment with peripheral anti-TNF-α reduced microglial activation, and cognitive and motor alterations in rats with HE [22, 23].

CCL2 has been demonstrated to play an important role in various neurodegenerative diseases. In the researches of Alzheimer’s disease (AD) and HIV dementia it was found that CCL2 could accelerate the decline of cognitive function, leading to the formation of dementia, indicating that CCL2 may be related to cognitive dysfunction [24, 25]. Moreover, increased CCL2 level in blood and cerebrospinal fluid have also been observed in patients with Parkinson’s disease (PD) [26], indicating that CCL2 may also be involved in the occurrence and development of PD. These findings prompted us to detect changes in the expression of CCL2 during HE. We found CCL2 concentration in cerebral cortex was significantly increased in rats with HE. The source of CCL2 was not determined in this work. However, it was identified by Matthew McMillin et al. that during HE, CCL2 was expressed in neurons [7].

Based on the fact that microglia activation and CCL2 expression were both increased during HE, it was interesting to investigate the effect of CCL2 on the activation of microglia. It was reported that CCL2 could attract microglia and promote the proliferation of microglia [8, 27]. In this work, we also demonstrated that neuron-derived CCL2 could induce the production of new microglia, which indicated during HE, CCL2 could increase microglia population by attracting these cells and accelerating their proliferation. In line with this work, it was found that CCL2 could recruit microglia, and promote its proliferation and production of inflammatory factors and oxygen free radicals in AD [28]. It was also demonstrated CCL2 was a key mediator of microglia activation in neuropathic pain states [29]. However, it was remained to be investigated whether CCL2 could promote microglia activation, neuroinflammation occurrence, and subsequent neurons death. It was observed that CCL2 knockout could suppress microglia activation during the first 24 h in a rats model of intracranial hemorrhage [30], suggesting CCL2 was involved in acute microglia activation. In this work, we found neuron-derived CCL2 induced classical activation of microglia in vitro and the production of proinflammatory factors, such as IL-6 and IL-1β.

Transcription factor NF-κB is the central substance of immune and inflammatory response and a transcriptional regulator in cell apoptosis signaling pathway, which plays an important role in the occurrence and development of HE [31]. It was reported that blocking the transcriptional activity of NF-κB could inhibit the expression of pro-inflammatory mediators, such as iNOS, COX2, IL-6, MCP-1 and TNF-α in microglia [32]. IκBα is an important inhibitor of NF-κB signaling pathway, which forms a complex with NF-κB p50 and NF-κBp65. This inhibits NF-κB nuclear translocation and regulation on transcription. Once IκBα is phosphorylated, it will dissociate from the complex. Then, NF-κB is imported into nucleus and combined with the motif to promote the transcription of the inflammation related genes. Our study found that the neuron-derived CCL2 could promote the phosphorylation of IκBα in microglia, and thus accelerate NF-κBp65 nuclear translocation. The role of CCL2 in the activation of NF-κB has been demonstrated in a variety of cells, such as human chondrosarcoma cells [33], dorsal root ganglion neurons [34], but to the best of our knowledge, this was the first report that CCL2 could induce the activation of NF-κB in microglia.

In addition to microglia activation, destruction of blood brain barrier (BBB) also plays an important role in the pathological process of HE. The permeability of BBB increased significantly in acute hepatic failure model inducing by D-galactosamine azoxymethane and hepatic devascularization [35, 36], which were attributed to the action of circulating toxic substances, such as ammonia, methyl octanoate, mercaptans, and phenol. In this study, in addition to increased brain CCL2 expression, the CCL2 in the circulation was also remarkably elevated, indicating that the integrity of BBB might be impaired in HE. It was previously demonstrated that CCL2 knockout could depress the decrement of BBB permeability in a rats model of stroke [37]. Moreover, CCL2 could improve the permeability of the BBB in vitro model composed of cerebral brain endothelial cell and astrocytes [38]. However, the effect of CCL2 on the BBB permeability during HE lacks direct evidences.

In summary, the present study suggest that following acute liver failure, CCL2 induced microglia activation and subsequent inflammatory cytokines release play an important role in neurological dysfunction during HE, and inhibition of CCL2 action using CCL2 receptor inhibitors, CCL2 neutralizing antibody or knockdown of CCL2 expression may be a potential therapeutic method for patients suffering from HE.

## Abbreviations

CCL2: chemokine CC motif ligand 2
HE: hepatic encephalopathy
TAA: thioacetamide
CNS: central nervous system
MCP-1: monocyte chemoattractant protein-1
ALT: aminotransferase
HRP: horseradish peroxidase
CM: culture medium
SD: standard deviation
